# Novel Dynamic Structures of 2019-nCoV with Nonlocal Operator via Powerful Computational Technique

**DOI:** 10.3390/biology9050107

**Published:** 2020-05-21

**Authors:** Wei Gao, P. Veeresha, D. G. Prakasha, Haci Mehmet Baskonus

**Affiliations:** 1 Faculty of Education, Harran University, Sanliurfa 63200, Turkey; gaowei@ynnu.edu.cn; 2 School of Information Science and Technology, Yunnan Normal University, Kunming 650500, China; 3 Department of Mathematics, Karnatak University, Dharwad 580003, India; viru0913@gmail.com; 4 Department of Mathematics, Davangere University, Shivagangothri, Davangere 577007, India; prakashadg@gmail.com

**Keywords:** coronavirus 2019-nCoV, Caputo fractional derivative, fractional natural decomposition method, RNA, numerical behavior

## Abstract

In this study, we investigate the infection system of the novel coronavirus (2019-nCoV) with a nonlocal operator defined in the Caputo sense. With the help of the fractional natural decomposition method (FNDM), which is based on the Adomian decomposition and natural transform methods, numerical results were obtained to better understand the dynamical structures of the physical behavior of 2019-nCoV. Such behaviors observe the general properties of the mathematical model of 2019-nCoV. This mathematical model is composed of data reported from the city of Wuhan, China.

## 1. Introduction

The world has been affected by a novel coronavirus pandemic, known as the 2019 novel coronavirus (2019-nCoV), which reportedly originated in Wuhan, central China [1]. It has been proposed that 2019-nCoV originated in the transmission from animal to human, as many of the initial infected patients claimed that they had been to a local fish and wild animal market in Wuhan in November [2]. Researchers soon confirmed that the disease is also transmitted from person to person [3]. According to data reported by the World Health Organization (WHO), by 21 March, 2020, there were more than 292,142 reported laboratory-confirmed human infections in 187 countries and territories around the world, including 12,784 cases resulting in death [4]. The death rate was also high in countries such as Italy and Spain. This confirms the severity and high infectivity of 2019-nCoV. Most people infected with 2019-nCoV experience mild to moderate respiratory illness, such as breathing difficulties, low fever, nausea, coughing and other symptoms. Some cases are asymptomatic. However, other symptoms, such as gastroenteritis and neurological diseases of varying severity, have also been reported [5]. 2019-nCoV is transmitted mainly through droplets from the nose when an infected person coughs or sneezes. Therefore, the best method to prevent the virus is to avoid meeting and touching other people. For this purpose, the Chinese government implemented a lockdown of the city of Wuhan and cut or limited transportation throughout China, including airplanes, trains, buses, and private cars, to limit the movement of the population. People were required to stay at home and to have their body temperatures taken each day, and were advised to wear masks or respirators if it was necessary for them to leave their homes. With the outbreak and transmission of 2019-nCoV around the world, other governments implemented similar measures, banning or imposing regulations on international travel, as well as closing schools, shopping malls, and companies. The 2019-nCoV pandemic has led to serious economic damage throughout the world, and has placed a strain on the administrative abilities of countries and their populations.

Many doctors and researchers have committed their expertise to the study of the virus. The novel coronavirus has been examined from various points of view, in many fields of study, including virology; infectious disease studies; microbiology; public, environmental and occupational health; veterinary science; sociology; media studies; politics and economics. In response to early outbreaks of the virus, China, USA, and Korea emerged as the leading countries in 2019-nCoV research.

Several researchers have studied the origins of 2019-nCoV. Initially, it was proposed that bats were the origin of 2019-nCoV, as was the case with Severe Acute Respiratory Syndrome (SARS), which caused epidemics in China and other regions of the world in 2003 [6,7]. Researchers have compared the 2019-nCoV outbreak with SARS and the Middle East Respiratory Syndrome (MERS) outbreak that occurred in 2012, with the goal of learning lessons from the two previous pandemics. According to Lu et al. [8], 2019-nCoV, like the viruses that cause SARS and MERS, belongs to the genus *Betacoronavirus*. According to Zhou, previous research indicates that 2019-nCoV displays a high level of similarity to the SARS coronavirus (SARS-CoV) based on full-length genome phylogenetic analysis, as well as the viruses’ putatively similar cell entry mechanisms and human cell receptor usage [9]. Xia et al., considering the high identity of receptor-binding domain (RBD) in 2019-nCoV and SARS-CoV, raised the idea that a SARS-CoV-specific human monoclonal antibody (CR3022) could bind potently with the RBD of 2019-nCoV (KD of 6.3 nM). This indicates that the difference in the RBD of SARS-CoV and 2019-nCoV has a crucial influence on the cross-reactivity of neutralizing antibodies, and that it is still necessary to develop novel monoclonal antibodies that could bind specifically to the RBD of 2019-nCoV [10]. Building on previous studies of the immunological system and structures of SARS-CoV, Syed et al. analyzed available, experimentally-determined, SARS-CoV-derived B-and T-cell epitopes and found that they are completely identical and comprise no mutation in the available 2019-nCoV sequence. This was a significant step in narrowing the search for potent targets for an effective vaccine against 2019-nCoV [11].

Other researchers focused on the transmission of 2019-nCoV among humans and the identification of transmissions. It is well accepted that human-to-human transmission has led to rapid growth in the number of infections. Rambaut claimed that after sequencing viral strains from a sample of infected people, little genetic variation was found, implying that the strains descended from a common ancestor [12]. Poon argued that sequences of the seven conserved viral replicase domains in the ORF1ab region show 94.6% identity between 2019-nCoV and SARS-CoV [13]. In the view of Chaudhury et al., computational protein–protein docking with accurate, physics-based energy functions is able to reveal the native-like, low-energy protein–protein complex from the unbound structures of two individual, interacting protein components [14]. Christians studied the transmission pattern of 2019-nCoV and found that the individual variation in the number of secondary cases provides further information about the early outbreak dynamics and the expected number of super-spreading events [15]. Huang C et al. have introduced held that the virus can also be spread through interspecies transmission [16]. Domenico et al. [17] built a phylogenetic tree using the 15 available whole genome sequences of 2019-nCoV and 12 highly similar sequences available in the gene bank (five from SARS, two from MERS, and five from bat SARS-like coronavirus). They held that 2019-nCoV was likely to have been transmitted from bats or another host, where mutations conferred upon it the ability to infect humans [17]. Other groups of researchers focused on virus prevention and the improvement of health care capacities, suggesting that the key to controlling virus transmission is the reduction of social distance and human contact through school closures, the shutting down of public transport, the suspension of common activities, etc. [18]. Others, like Koonin and Cetron, asserted that case isolation, household quarantine, and internal travel restrictions are also necessary for virus control [19]. Therefore, many mathematical properties of real world problems including fractional or integer order have been introduced to better understanding of deeper properties and to analyze by many researchers [20,21,22,23,24,25,26,27,28,29,30,31,32,33,34,35,36,37,38,39,40,41,42,43,44,45,46,47,48,49,50,51,52,53,54,55,56,57,58,59,60,61,62,63,64,65,66,67,68].

We aimed to understand and investigate the 2019-nCoV infection system from the perspective of mathematics. The fractional natural decomposition method (FNDM), which is based on decomposition and natural transformation, was employed to obtain numerical results that may help to understand the dynamical structures of the physical behavior of 2019-nCoV. The model is defined by a system of six equations, illustrating the outbreak of the coronavirus in the form of nonlinear ordinary differential equations. Susceptible people are expressed as $S_{p}\left(t\right)$, the exposed population is expressed as $E_{p}\left(t\right)$, total infected is $I_{p}\left(t\right)$, asymptotically-infected population is $A_{p}\left(t\right)$, the total number of humans recovered is $R_{p}\left(t\right)$, reservoir is M(t), and their corresponding interaction is presented as follows [20,64]:

(1) $$\begin{array}{l} \frac{dS_{p}\left(t\right)}{dt}=n_{p}-m_{p}S_{p}-b_{p}S_{p}\left(I_{p}+\kappa A_{p}\right)-b_{w}S_{p}M,\\ \frac{dE_{p}\left(t\right)}{dt}=b_{p}S_{p}\left(I_{p}+\kappa A_{p}\right)+b_{w}S_{p}M_{p}-\left(1-\delta_{p}\right)\omega_{p}E_{p}-\delta_{p}\omega_{p}^{\prime }E_{p}-m_{p}E_{p},\\ \frac{dI_{p}\left(t\right)}{dt}=\left(1-\delta_{p}\right)\omega_{p}E_{p}-\left(\gamma_{p}+m_{p}\right)I_{p},\\ \frac{dA_{p}\left(t\right)}{dt}=\delta_{p}\omega_{p}^{\prime }E_{p}-\left(\gamma_{p}^{\prime }+m_{p}\right)A_{p},\\ \frac{dR_{p}\left(t\right)}{dt}=\gamma_{p}I_{p}+\gamma_{p}^{\prime }A_{p}-m_{p}R_{p},\\ \frac{dM\left(t\right)}{dt}=\varepsilon I_{p}+\sigma A_{p}-\vartheta M, \end{array}$$

where $n_{p}$ denotes the rate of birth and $m_{p}$ denotes the rate of death in the infected population; $b_{p}$ represents the transmission coefficient; $b_{w}$ is the disease transmission coefficient; κ is the transmissibility multiple; $\omega_{p}$ and $\omega_{p}^{\prime }$ signify the incubation period; $\gamma_{p}$ and $\gamma_{p}^{\prime }$ are the recovery rate of $I_{p}$ and $A_{p}$, respectively; ε and σ denote the influence of the virus from $I_{p}$ and $A_{p}$ to M, respectively; and ϑ represents the rate of eliminating the virus from M. For further discussion of these parameters, refer to and the corresponding values of the parameters were taken from [20,64]. The process of estimating the equilibrium point and the disease-free equilibrium of the considered model is important because it can help predict the behavior and future of the model. The basic reproduction number helps to understand future evolution and to determine what proportion of the population should be immunized through vaccination in order to eradicate the disease.

In this manuscript, we consider the generalization of the above model with the help of fractional calculus. The model is generalized to incorporate memory consequences and hereditary properties. This builds upon the interesting results illustrated and confirmed by Ionescu et al. and Qureshi [62,63] concerning the usefulness of fractional order operators in biological models. Ionescu et al. illustrated the importance of the fractional operator in drug diffusion, neuroscience, bio-impedance, and respiratory tissue and structure [62]. The authors in [63] showed the importance of the Caputo fractional derivative while studying the autonomous dynamical system of measles. Many researchers have used the fractional operator to study various epidemic models. Equation (1) may be reconsidered in the sense of Caputo as following:

(2) $$\begin{array}{l} D_{t}^{\alpha }S_{p}\left(t\right)=n_{p}-m_{p}S_{p}-b_{p}S_{p}\left(I_{p}+\kappa A_{p}\right)-b_{w}S_{p}M,\\ D_{t}^{\alpha }E_{p}\left(t\right)=b_{p}S_{p}\left(I_{p}+\kappa A_{p}\right)+b_{w}S_{p}M_{p}-\left(1-\delta_{p}\right)\omega_{p}E_{p}-\delta_{p}\omega_{p}^{\prime }E_{p}-m_{p}E_{p},\\ D_{t}^{\alpha }I_{p}\left(t\right)=\left(1-\delta_{p}\right)\omega_{p}E_{p}-\left(\gamma_{p}+m_{p}\right)I_{p},\\ D_{t}^{\alpha }A_{p}\left(t\right)=\delta_{p}\omega_{p}^{\prime }E_{p}-\left(\gamma_{p}^{\prime }+m_{p}\right)A_{p},\\ D_{t}^{\alpha }R_{p}\left(t\right)=\gamma_{p}I_{p}+\gamma_{p}^{\prime }A_{p}-m_{p}R_{p},\\ D_{t}^{\alpha }M\left(t\right)=\varepsilon I_{p}+\sigma A_{p}-\vartheta M, \end{array}$$

where α is in the Caputo sense.

## 2. Preliminaries

In this section, we recall some fundamentals of fractional calculus.

**Definition** **1.** *In the fractional Riemann–Liouville sense, the integral of a function*$f\left(t\right)\in C_{\delta }\left(\delta \geq -1\right)$*is presented as:*

(3) $$\begin{array}{l} J^{\alpha }f\left(t\right)=\frac{1}{\Gamma \left(\mu \right)}\int_{0}^{t}{\left(t-\vartheta \right)}^{\mu -1}f\left(\vartheta \right)d\vartheta ,\\ J^{0}f\left(t\right)=f\left(t\right), \end{array}$$

*in which*$C_{\delta }$ is continuous function domain.

**Definition** **2.** *The Caputo fractional derivative of* $f\in C_{-1}^{n}$ *is presented as:*

(4) $$D_{t}^{\alpha }f\left(t\right)=\{\begin{array}{c} \frac{d^{n}f\left(t\right)}{dt^{n}},\alpha =n\in \mathbb{N},\\ \frac{1}{\Gamma \left(n-\alpha \right)}\int_{0}^{t}{\left(t-\vartheta \right)}^{n-\alpha -1}f^{\left(n\right)}\left(\vartheta \right)d\vartheta ,n-1<\alpha <n,n\in \mathbb{N}, \end{array}$$

*where* $C_{-1}^{n}$ *is continuousfunction domain.*

**Definition** **3.** *The Mittag–Leffler type function with one-parameter is defined* [21] *as:*

(5) $$E_{\alpha }\left(z\right)=\sum_{k=0}^{\infty }\frac{z^{k}}{\Gamma \left(\alpha k+1\right)},\;\alpha >0,\;z\in \mathbb{C}.$$

**Definition** **4.** *The natural transform (NT) of *f(t)*is symbolized by *ℕ[f(t)]*for* t∈ℝ*and presented with the NT variables *s*and*W by [22]:

$$\mathbb{N}\left[f\left(t\right)\right]=R\left(s,W\right)=\int_{-\infty }^{\infty }e^{-st}f\left(\omega t\right)dt;\;s,W\in \left(-\infty ,\infty \right)$$.

*We define the NT with the Heaviside function* H(t) as

(6) $$\mathbb{N}\left[f\left(t\right)H\left(t\right)\right]={\mathbb{N}}^{+}\left[f\left(t\right)\right]=R^{+}\left(s,W\right)\;=\int_{0}^{\infty }e^{-st}f\left(W,\;t\right)dt;\;s,W\in \left(0,\infty \right)\;\mathrm{and}\;t\in \mathbb{R}.$$

*For* W=1 *, Equation (6) is reduced to the Laplace transform and for* s=1 *, Equation (6) represents the Sumudu transform.*

**Theorem** **1.** *The NT*$\;R_{\alpha }\left(s,\;\omega \right)$ *of the fractional derivative of *f(t) *Riemann–Liouville sense is symbolized by* $D^{\alpha }f\left(t\right)$ *and defined as [22]:*

(7) $${\mathbb{N}}^{+}\left[D^{\alpha }f\left(t\right)\right]=R_{\alpha }\left(s,\;W\right)=\frac{s^{\alpha }}{\omega^{\alpha }}R\left(s,W\right)-\sum_{k=0}^{n-1}\frac{s^{k}}{W^{\alpha -k}}{\left[D^{\alpha -k-1}f\left(t\right)\right]}_{t=0},$$

*where* R(s, W) *is NT of* f(t), α *is the order and* n *is any positive integer.* n−1≤α<n.

**Theorem** **2.** *The natural transform* $R_{\alpha }\left(s,\;W\right)$ *of the arbitrary derivative in the Caputo sense of *f(t) *is symbolized by* Dcαf(t)*and defined as [23]:*

(8) ℕ+[Dcαf(t)]=Rαc(s, W)=sαωαR(s,W)−∑k=0n−1sα−(k+1)Wα−k[Dkf(t)]t=0.

**Remark** **1.** *Some basic properties of the NT are defined:*

(i) ${\mathbb{N}}^{+}\left[1\right]=\frac{1}{s},$
(ii) ${\mathbb{N}}^{+}\left[t^{\alpha }\right]=\frac{\Gamma \left(\alpha +1\right)W^{\alpha }}{s^{\alpha +1}},$
(iii) ${\mathbb{N}}^{+}\left[f^{\left(n\right)}\left(t\right)\right]=\frac{s^{n}}{W^{n}}R\left(s,\;W\right)-\overset{n-1}{\underset{k=0}{\sum }}\frac{s^{n-\left(k+1\right)}}{W^{n-k}}\frac{\Gamma \left(\alpha +1\right)W^{\alpha }}{s^{\alpha +1}}.$

## 3. Method of Solution for the Projected System

It is essential use an efficient technique to find the solution for the projected system. Recently, many analytical and numerical schemes have been suggested for solving linear and nonlinear differential equations. When examining the distinct class of real-world problems, many researchers have acknowledged the limitations of well-established schemes. In this study, we used a powerful algorithm capable of solving a nonlinear model with partial differential equations without converting them to ordinary differential equations. This method of solution does not require any perturbation or dissertation. It is a mixture of the Adomian decomposition technique [24] and natural transformation [25] proposed by Rawashdeh et al. [26,27].

Here, we consider a coupled system to illustrate the basic solution procedure of the considered algorithm with initial conditions

(9) $$\begin{array}{l} D_{t}^{\alpha }u\left(x,t\right)+Ru\left(x,\;t\right)+Fu\left(x,\;t\right)=h_{1}\left(x,t\right),\\ D_{t}^{\alpha }v\left(x,t\right)+Rv\left(x,\;t\right)+Fv\left(x,t\right)=h_{2}\left(x,t\right), \end{array}$$

and

(10) $$u\left(x,0\right)=g_{1}\left(x\right),\;v\left(x,0\right)=g_{2}\left(x\right),$$

where $D^{\alpha }u\left(x,t\right)$ and $D^{\alpha }v\left(x,t\right)$ signify the fractional Caputo derivatives of u(x,t) and v(x,t), respectively; $h_{1}\left(x,t\right)$ and $h_{2}\left(x,t\right)$ are the source terms; and F and R denote the nonlinear and linear differential operators, respectively. On applying *NT* and with the help of Theorem 2, then Equation (9) produces:

(11) $$\begin{array}{l} U\left(x,s,W\right)=\frac{u^{\alpha }}{s^{\alpha }}\overset{n-1}{\underset{k=0}{\sum }}\frac{s^{\alpha -\left(k+1\right)}}{W^{\alpha -k}}{\left[D^{k}u\left(x,t\right)\right]}_{t=0}+\frac{W^{\alpha }}{s^{\alpha }}{\mathbb{N}}^{+}\left[h_{1}\left(x,t\right)\right]\\ -\frac{W^{\alpha }}{s^{\alpha }}{\mathbb{N}}^{+}\left[Rv\left(x,,t\right)+Fu\left(x,t\right)\right],\\ V\left(x,s,W\right)=\frac{v^{\alpha }}{s^{\alpha }}\overset{n-1}{\underset{k=0}{\sum }}\frac{s^{\alpha -\left(k+1\right)}}{W^{\alpha -k}}{\left[D^{k}v\left(x,t\right)\right]}_{t=0}+\frac{W^{\alpha }}{s^{\alpha }}{\mathbb{N}}^{+}\left[h_{2}\left(x,t\right)\right]\\ -\frac{W^{\alpha }}{s^{\alpha }}{\mathbb{N}}^{+}\left[Ru\left(x,,t\right)+Fv\left(x,t\right)\right]. \end{array}$$

On employing inverse NT on Equation (11), we obtain:

(12) $$u\left(x,t\right)=G\left(x,t\right)-{\mathbb{N}}^{-1}\left[\frac{W^{\alpha }}{s^{\alpha }}{\mathbb{N}}^{+}\left[Rv\left(x,\;t\right)+Fu\left(x,t\right)\right]\right],\;v\left(x,t\right)=H\left(x,t\right)-{\mathbb{N}}^{-1}\left[\frac{W^{\alpha }}{s^{\alpha }}{\mathbb{N}}^{+}\left[Ru\left(x,t\right)+Fv\left(x,t\right)\right]\right].$$

From given initial conditions, non-homogeneous terms G(x,t) and H(x,t) exist. The infinite series solution is presented as:

(13) $$u\left(x,t\right)=\overset{\infty }{\underset{n=0}{\sum }}u_{n}\left(x,t\right),Fu\left(x,t\right)=\overset{\infty }{\underset{n=0}{\sum }}A_{n},\;v\left(x,t\right)=\overset{\infty }{\underset{n=0}{\sum }}v_{n}\left(x,t\right),Fv\left(x,t\right)=\overset{\infty }{\underset{n=0}{\sum }}B_{n},$$

where the $A_{n}$ and $\;B_{n}$ indicate the nonlinear terms of Fu(x, t) and Fv(x,t), respectively. Using Equations (12) and (13), we obtain

(14) $$\overset{\infty }{\underset{n=0}{\sum }}u_{n}\left(x,t\right)=G\left(x,t\right)-{\mathbb{N}}^{-1}\left[\frac{W^{\alpha }}{s^{\alpha }}{\mathbb{N}}^{+}\left[R\overset{\infty }{\underset{n=0}{\sum }}v_{n}\left(x,t\right)\right]+\overset{\infty }{\underset{n=0}{\sum }}A_{n}\right],\;\overset{\infty }{\underset{n=0}{\sum }}v_{n}\left(x,t\right)=H\left(x,t\right)-{\mathbb{N}}^{-1}\left[\frac{W^{\alpha }}{s^{\alpha }}{\mathbb{N}}^{+}\left[R\overset{\infty }{\underset{n=0}{\sum }}u_{n}\left(x,t\right)\right]+\overset{\infty }{\underset{n=0}{\sum }}B_{n}\right].$$

By comparing both sides of Equation (14), we obtain:

$u_{0}\left(x,t\right)=G\left(x,t\right),$

$u_{1}\left(x,t\right)=-{\mathbb{N}}^{-1}\left[\frac{W^{\alpha }}{s^{\alpha }}{\mathbb{N}}^{+}\left[Rv_{0}\left(x,t\right)\right]+A_{0}\right],$

$u_{2}\left(x,t\right)=-{\mathbb{N}}^{-1}\left[\frac{W^{\alpha }}{s^{\alpha }}{\mathbb{N}}^{+}\left[Rv_{1}\left(x,t\right)\right]+A_{1}\right],$

⋮

$v_{0}\left(x,t\right)=H\left(x,t\right),$

$v_{1}\left(x,t\right)=-{\mathbb{N}}^{-1}\left[\frac{W^{\alpha }}{s^{\alpha }}{\mathbb{N}}^{+}\left[Ru_{0}\left(x,t\right)\right]+B_{0}\right],$

$v_{2}\left(x,t\right)=-{\mathbb{N}}^{-1}\left[\frac{W^{\alpha }}{s^{\alpha }}{\mathbb{N}}^{+}\left[Ru_{1}\left(x,t\right)\right]+B_{1}\right],$

⋮

Similarly, we can obtain the recursive relation in general form for n≥1 and defined as:

(15) $$u_{n+1}\left(x,t\right)=-{\mathbb{N}}^{-1}\left[\frac{W^{\alpha }}{s^{\alpha }}{\mathbb{N}}^{+}\left[Rv_{n}\left(x,t\right)\right]+A_{n}\right],\;v_{n+1}\left(x,t\right)=-{\mathbb{N}}^{-1}\left[\frac{W^{\alpha }}{s^{\alpha }}{\mathbb{N}}^{+}\left[Ru_{n}\left(x,t\right)\right]+B_{n}\right].$$

Lastly, the approximate solutions are defined as follows:

$u\left(x,t\right)=\sum_{n=0}^{\infty }u_{n}\left(x,t\right),v\left(x,t\right)=\sum_{n=0}^{\infty }v_{n}\left(x,t\right).$

## 4. FNDM Solution for the Projected System

Here, we consider the 2019-nCoV system of equations, presented in Equation (2), and find the solution using the projected solution procedure. Then,

(16) $$\left\{ \begin{array}{c} D_{t}^{\alpha }S_{p}\left(t\right)=n_{p}-m_{p}S_{p}-b_{p}S_{p}(I_{p}+\kappa A_{p})-b_{w}S_{p}M,\\ D_{t}^{\alpha }E_{p}\left(t\right)=b_{p}S_{p}\left(I_{p}+\kappa A_{p}\right)+b_{w}S_{p}M_{p}-\left(1-\delta_{p}\right)\omega_{p}E_{p}-\delta_{p}\omega_{p}^{'}E_{p}-m_{p}E_{p},\\ D_{t}^{\alpha }I_{p}\left(t\right)=\left(1-\delta_{p}\right)\omega_{p}E_{p}-\left(\gamma_{p}+m_{p}\right)I_{p},\\ D_{t}^{\alpha }A_{p}\left(t\right)=\delta_{p}\omega_{p}^{'}E_{p}-\left(\gamma_{p}^{'}+m_{p}\right)A_{p},\\ D_{t}^{\alpha }R_{p}\left(t\right)=\gamma_{p}I_{p}+\gamma_{p}^{'}A_{p}-m_{p}R_{p},\\ D_{t}^{\alpha }M\left(t\right)=\varepsilon I_{p}+\sigma A_{p}-\vartheta M,\\ 0<\alpha \leq 1 \end{array}\right.$$

associated to initial conditions produces

(17) $$S\left(0\right)=S_{0},\;E\left(0\right)=E_{0},\;I\left(0\right)=I_{0},\;A\left(0\right)=A_{0},\;R\left(0\right)=R_{0},\;M\left(0\right)=M_{0}.$$

With the assistance of NT on Equation (16), we obtain:

(18) $$\begin{array}{l} {\mathbb{N}}^{+}\left[D_{t}^{\alpha }S_{p}\left(t\right)\right]={\mathbb{N}}^{+}\left[n_{p}-m_{p}S_{p}-b_{p}S_{p}\left(I_{p}+\kappa A_{p}\right)-b_{w}S_{p}M\right],\\ {\mathbb{N}}^{+}\left[D_{t}^{\alpha }E_{p}\left(t\right)\right]={\mathbb{N}}^{+}\left[b_{p}S_{p}\left(I_{p}+\kappa A_{p}\right)+b_{w}S_{p}M_{p}-\left(1-\delta_{p}\right)\omega_{p}E_{p}-\delta_{p}\omega_{p}^{\prime }E_{p}-m_{p}E_{p}\right],\\ {\mathbb{N}}^{+}\left[D_{t}^{\alpha }I_{p}\left(t\right)\right]={\mathbb{N}}^{+}\left[\left(1-\delta_{p}\right)\omega_{p}E_{p}-\left(\gamma_{p}+m_{p}\right)I_{p}\right],\\ {\mathbb{N}}^{+}\left[D_{t}^{\alpha }A_{p}\left(t\right)\right]={\mathbb{N}}^{+}\left[\delta_{p}\omega_{p}^{\prime }E_{p}-\left(\gamma_{p}^{\prime }+m_{p}\right)A_{p}\right],\\ {\mathbb{N}}^{+}\left[D_{t}^{\alpha }R_{p}\left(t\right)\right]={\mathbb{N}}^{+}\left[\gamma_{p}I_{p}+\gamma_{p}^{\prime }A_{p}-m_{p}R_{p}\right],\\ {\mathbb{N}}^{+}\left[D_{t}^{\alpha }M\left(t\right)\right]={\mathbb{N}}^{+}\left[\varepsilon I_{p}+\sigma A_{p}-\vartheta M\right]. \end{array}$$

The nonlinear operator is defined as:

(19) $$\begin{array}{l} \frac{s^{\alpha }}{W^{\alpha }}{\mathbb{N}}^{+}\left[S_{p}\left(t\right)\right]-\overset{n-1}{\underset{k=0}{\sum }}\frac{s^{\alpha -\left(k+1\right)}}{W^{\alpha -k}}{\left[D^{k}S_{p}\right]}_{t=0}={\mathbb{N}}^{+}\left[n_{p}-m_{p}S_{p}-b_{p}S_{p}\left(I_{p}+\kappa A_{p}\right)-b_{w}S_{p}M_{p}\right],\\ \frac{s^{\alpha }}{W^{\alpha }}{\mathbb{N}}^{+}\left[E_{p}\left(t\right)\right]-\overset{n-1}{\underset{k=0}{\sum }}\frac{s^{\alpha -\left(k+1\right)}}{W^{\alpha -k}}{\left[D^{k}E_{p}\right]}_{t=0}={\mathbb{N}}^{+}[b_{p}S_{p}\left(I_{p}+\kappa A_{p}\right)+b_{w}S_{p}M\\ -\left(1-\delta_{p}\right)\omega_{p}E_{p}-\delta_{p}\omega_{p}^{\prime }E_{p}-m_{p}E_{p}],\\ \frac{s^{\alpha }}{W^{\alpha }}{\mathbb{N}}^{+}\left[I_{p}\left(t\right)\right]-\overset{n-1}{\underset{k=0}{\sum }}\frac{s^{\alpha -\left(k+1\right)}}{W^{\alpha -k}}{\left[D^{k}I_{p}\right]}_{t=0}={\mathbb{N}}^{+}\left[\left(1-\delta_{p}\right)\omega_{p}E_{p}-\left(\gamma_{p}+m_{p}\right)I_{p}\right],\\ \frac{s^{\alpha }}{W^{\alpha }}{\mathbb{N}}^{+}\left[A_{p}\left(t\right)\right]-\overset{n-1}{\underset{k=0}{\sum }}\frac{s^{\alpha -\left(k+1\right)}}{W^{\alpha -k}}{\left[D^{k}A_{p}\right]}_{t=0}={\mathbb{N}}^{+}\left[\delta_{p}\omega_{p}^{\prime }E_{p}-\left(\gamma_{p}^{\prime }+m_{p}\right)A_{p}\right],\\ \frac{s^{\alpha }}{W^{\alpha }}{\mathbb{N}}^{+}\left[R_{p}\left(t\right)\right]-\overset{n-1}{\underset{k=0}{\sum }}\frac{s^{\alpha -\left(k+1\right)}}{W^{\alpha -k}}{\left[D^{k}R_{p}\right]}_{t=0}={\mathbb{N}}^{+}\left[\gamma_{p}I_{p}+\gamma_{p}^{\prime }A_{p}-m_{p}R_{p}\right],\\ \frac{s^{\alpha }}{W^{\alpha }}{\mathbb{N}}^{+}\left[M\left(t\right)\right]-\overset{n-1}{\underset{k=0}{\sum }}\frac{s^{\alpha -\left(k+1\right)}}{W^{\alpha -k}}{\left[D^{k}M\right]}_{t=0}={\mathbb{N}}^{+}\left[\varepsilon I_{p}+\sigma A_{p}-\vartheta M\right]. \end{array}$$

By the above equation, we obtain:

(20) $$\begin{array}{l} {\mathbb{N}}^{+}\left[S_{p}\left(t\right)\right]=\frac{1}{s}\left[S_{0}\right]+\frac{W^{\alpha }}{s^{\alpha }}{\mathbb{N}}^{+}\left[n_{p}-m_{p}S_{p}-b_{p}S_{p}\left(I_{p}+\kappa A_{p}\right)-b_{w}S_{p}M\right],\\ {\mathbb{N}}^{+}\left[E_{p}\left(t\right)\right]=\frac{1}{s}\left[E_{0}\right]+\frac{W^{\alpha }}{s^{\alpha }}{\mathbb{N}}^{+}\left[b_{p}S_{p}\left(I_{p}+\kappa A_{p}\right)+b_{w}S_{p}M_{p}-\left(1-\delta_{p}\right)\omega_{p}E_{p}-\delta_{p}\omega_{p}^{\prime }E_{p}-m_{p}E_{p}\right],\\ {\mathbb{N}}^{+}\left[I_{p}\left(t\right)\right]=\frac{1}{s}\left[I_{0}\right]+\frac{W^{\alpha }}{s^{\alpha }}{\mathbb{N}}^{+}\left[\left(1-\delta_{p}\right)\omega_{p}E_{p}-\left(\gamma_{p}+m_{p}\right)I_{p}\right],\\ {\mathbb{N}}^{+}\left[A_{p}\left(t\right)\right]=\frac{1}{s}\left[A_{0}\right]+\frac{W^{\alpha }}{s^{\alpha }}{\mathbb{N}}^{+}\left[\delta_{p}\omega_{p}^{\prime }E_{p}-\left(\gamma_{p}^{\prime }+m_{p}\right)A_{p}\right],\\ {\mathbb{N}}^{+}\left[R_{p}\left(t\right)\right]=\frac{1}{s}\left[R_{0}\right]+\frac{W^{\alpha }}{s^{\alpha }}{\mathbb{N}}^{+}\left[\gamma_{p}I_{p}+\gamma_{p}^{\prime }A_{p}-m_{p}R_{p}\right],\\ {\mathbb{N}}^{+}\left[M\left(t\right)\right]=\frac{1}{s}\left[M_{0}\right]+\frac{W^{\alpha }}{s^{\alpha }}{\mathbb{N}}^{+}\left[\varepsilon I_{p}+\sigma A_{p}-\vartheta M\right]. \end{array}$$

On employing inverse NT on Equation (20), we obtain:

(21) $$S_{p}\left(t\right)=S_{0}+{\mathbb{N}}^{-1}\left[\frac{W^{\alpha }}{s^{\alpha }}{\mathbb{N}}^{+}\left[n_{p}-m_{p}S_{p}-b_{p}S_{p}\left(I_{p}+\kappa A_{p}\right)-b_{w}S_{p}M\right]\right],\;E_{p}\left(t\right)=E_{0}+{\mathbb{N}}^{-1}\left[\frac{W^{\alpha }}{s^{\alpha }}{\mathbb{N}}^{+}\left[b_{p}S_{p}\left(I_{p}+\kappa A_{p}\right)+b_{w}S_{p}M_{p}-\left(1-\delta_{p}\right)\omega_{p}E_{p}-\delta_{p}\omega_{p}^{\prime }E_{p}-m_{p}E_{p}\right]\right],\;I_{p}\left(t\right)=I_{0}+{\mathbb{N}}^{-1}\left[\frac{W^{\alpha }}{s^{\alpha }}{\mathbb{N}}^{+}\left[\left(1-\delta_{p}\right)\omega_{p}E_{p}-\left(\gamma_{p}+m_{p}\right)I_{p}\right]\right],\;A_{p}\left(t\right)=A_{0}+{\mathbb{N}}^{-1}\left[\frac{W^{\alpha }}{s^{\alpha }}{\mathbb{N}}^{+}\left[\delta_{p}\omega_{p}^{\prime }E_{p}-\left(\gamma_{p}^{\prime }+m_{p}\right)A_{p}\right]\right],\;R_{p}\left(t\right)=R_{0}+{\mathbb{N}}^{-1}\left[\frac{W^{\alpha }}{s^{\alpha }}{\mathbb{N}}^{+}\left[\gamma_{p}I_{p}+\gamma_{p}^{\prime }A_{p}-m_{p}R_{p}\right]\right],\;M\left(t\right)=M_{0}+{\mathbb{N}}^{-1}\left[\frac{W^{\alpha }}{s^{\alpha }}{\mathbb{N}}^{+}\left[\varepsilon I_{p}+\sigma A_{p}-\vartheta M\right]\right].$$

Let us consider the series solution for $S_{p}\left(t\right),E_{p}\left(t\right),I_{p}\left(t\right),A_{p}\left(t\right),R_{p}\left(t\right)$, and M(t) respectively as follows:

$S_{p}\left(t\right)=\overset{\infty }{\underset{n=0}{\sum }}{S_{p}}_{n}\left(t\right),\;E\left(t\right)=\overset{\infty }{\underset{n=0}{\sum }}{E_{p}}_{n}\left(t\right),$

$I\left(t\right)=\overset{\infty }{\underset{n=0}{\sum }}{I_{p}}_{n}\left(t\right),\;A\left(t\right)=\overset{\infty }{\underset{n=0}{\sum }}{A_{p}}_{n}\left(t\right),\;$

$R\left(t\right)=\overset{\infty }{\underset{n=0}{\sum }}{R_{p}}_{n}\left(t\right),\;M\left(t\right)=\overset{\infty }{\underset{n=0}{\sum }}M\left(t\right).$

Note that $S_{p}I_{p}=\sum_{n=0}^{\infty }P_{1,n},S_{p}A_{p}=\sum_{n=0}^{\infty }P_{2,n}$, and $S_{p}M=\sum_{n=0}^{\infty }P_{3,n}$ represent the nonlinear terms and are known as the Adomian polynomials. With the help of these terms, Equation (21) becomes:

(22) $$\begin{array}{l} \overset{\infty }{\underset{n=0}{\sum }}{S_{p}}_{n}\left(t\right)=S_{0}+{\mathbb{N}}^{-1}\left[\frac{W^{\alpha }}{s^{\alpha }}{\mathbb{N}}^{+}\left[n_{p}-m_{p}{S_{p}}_{n}-b_{p}\overset{\infty }{\underset{n=0}{\sum }}P_{1,n}-\kappa b_{p}\overset{\infty }{\underset{n=0}{\sum }}P_{2,n}-b_{w}\overset{\infty }{\underset{n=0}{\sum }}P_{3,n}\right]\right],\\ \overset{\infty }{\underset{n=0}{\sum }}{E_{p}}_{n}\left(t\right)=E_{0}+{\mathbb{N}}^{-1}\left[\frac{W^{\alpha }}{s^{\alpha }}{\mathbb{N}}^{+}\left[b_{p}\overset{\infty }{\underset{n=0}{\sum }}P_{1,n}+\kappa b_{p}\overset{\infty }{\underset{n=0}{\sum }}P_{2,n}+b_{w}\overset{\infty }{\underset{n=0}{\sum }}P_{3,n}-\left(\left(1-\delta_{p}\right)\omega_{p}+\delta_{p}\omega_{p}^{\prime }+m_{p}\right){E_{p}}_{n}\right]\right],\\ \overset{\infty }{\underset{n=0}{\sum }}{I_{p}}_{n}\left(t\right)=I_{0}+{\mathbb{N}}^{-1}\left[\frac{W^{\alpha }}{s^{\alpha }}{\mathbb{N}}^{+}\left[\left(1-\delta_{p}\right)\omega_{p}{E_{p}}_{n}-\left(\gamma_{p}+m_{p}\right){I_{p}}_{n}\right]\right],\\ \overset{\infty }{\underset{n=0}{\sum }}{A_{p}}_{n}\left(t\right)=A_{0}+{\mathbb{N}}^{-1}\left[\frac{W^{\alpha }}{s^{\alpha }}{\mathbb{N}}^{+}\left[\delta_{p}\omega_{p}^{\prime }{E_{p}}_{n}-\left(\gamma_{p}^{\prime }+m_{p}\right){A_{p}}_{n}\right]\right],\\ \overset{\infty }{\underset{n=0}{\sum }}{R_{p}}_{n}\left(t\right)=R_{0}+{\mathbb{N}}^{-1}\left[\frac{W^{\alpha }}{s^{\alpha }}{\mathbb{N}}^{+}\left[\gamma_{p}{I_{p}}_{n}+\gamma_{p}^{\prime }{A_{p}}_{n}-m_{p}{R_{p}}_{n}\right]\right],\\ \overset{\infty }{\underset{n=0}{\sum }}M_{n}\left(t\right)=M_{0}+{\mathbb{N}}^{-1}\left[\frac{W^{\alpha }}{s^{\alpha }}{\mathbb{N}}^{+}\left[\varepsilon {I_{p}}_{n}+\sigma {A_{p}}_{n}-\vartheta M_{n}\right]\right]. \end{array}$$

With the assistance of the above system with prescribed initial conditions, we find the terms of the series solution for the projected model systematically. Then, we establish the series solutions as for the second iterations:

$$S_{p}\left(t\right)=\overset{\infty }{\underset{n=0}{\sum }}{S_{p}}_{n}\left(t\right)=S_{0}+{S_{p}}_{1}\left(t\right)+{S_{p}}_{2}\left(t\right)+\cdots \;=S_{0}+\frac{t^{\alpha }}{\Gamma \left[\alpha +1\right]}\left(n_{p}-\kappa A_{0}b_{p}S_{0}-m_{p}S_{0}-b_{w}M_{0}S_{0}-b_{p}I_{0}S_{0}\right)+\frac{t^{\alpha }}{\Gamma \left[2\alpha +1\right]}(n_{p}(\Gamma \left[1+\alpha \right]\;-t^{\alpha }\kappa A_{0}b_{p}-t^{\alpha }b_{p}I_{0})+S_{0}(t^{\alpha }\kappa^{2}A_{0}^{2}b_{p}^{2}-2\Gamma \left[\alpha +1\right]b_{w}M_{0}+t^{\alpha }b_{p}b_{w}M_{0}I_{0}\;+t^{\alpha }b_{p}^{2}Q_{0}^{2}-m_{p}\left(\Gamma \left[\alpha +1\right]-2t^{\alpha }b_{p}I_{0}\right)+t^{\alpha }b_{p}I_{0}\gamma_{p}+t^{\alpha }\kappa A_{0}b_{p}(2m_{p}+b_{w}M_{0}\;+2b_{p}I_{0}+\gamma_{p})-t^{\alpha }b_{p}E_{0}\omega_{p}+t^{\alpha }b_{p}E_{0}\delta_{p}\omega_{p}-t^{\alpha }\kappa b_{p}E_{0}\delta_{p}\omega_{p}^{\prime }))+\cdots ,$$

$$E_{p}\left(t\right)=\overset{\infty }{\underset{n=0}{\sum }}{E_{p}}_{n}\left(t\right)=E_{0}+{E_{p}}_{1}\left(t\right)+{E_{p}}_{2}\left(t\right)+\cdots \;=E_{0}+\frac{t^{\alpha }}{\Gamma \left[\alpha +1\right]}(-m_{p}E_{0}+\kappa A_{0}b_{p}S_{0}+b_{w}M_{0}S_{0}+b_{p}I_{0}S_{0}+E_{0}\left(-1+\delta_{p}\right)\omega_{p}\;-E_{0}\delta_{p}\omega_{p}^{\prime })-\frac{t^{2\alpha }}{\Gamma \left[1+2\alpha \right]}(-m_{p}^{2}E_{0}-b_{p}n_{p}I_{0}+\kappa^{2}A_{0}^{2}b_{p}^{2}S_{0}+b_{w}^{2}M_{0}^{2}S_{0}+3b_{p}m_{p}I_{0}S_{0}\;+b_{p}^{2}Q_{0}^{2}S_{0}+b_{p}I_{0}S_{0}\gamma_{p}-2m_{p}E_{0}\omega_{p}-b_{p}E_{0}S_{0}\omega_{p}+b_{p}I_{0}S_{0}\omega_{p}+2m_{p}E_{0}\delta_{p}\omega_{p}\;+b_{p}E_{0}S_{0}\delta_{p}\omega_{p}-b_{p}I_{0}S_{0}\delta_{p}\omega_{p}-E_{0}\omega_{p}^{2}+2E_{0}\delta_{p}\omega_{p}^{2}-E_{0}\delta_{p}^{2}\omega_{p}^{2}-2m_{p}E_{0}\delta_{p}\omega_{p}^{\prime }\;-\kappa b_{p}E_{0}S_{0}\delta_{p}\omega_{p}^{\prime }+b_{p}I_{0}S_{0}\delta_{p}\omega_{p}^{\prime }-E_{0}\delta_{p}^{2}\omega_{p}^{2z}-2E_{0}\delta_{p}\omega_{p}^{1+z}+2E_{0}\delta_{p}^{2}\omega_{p}^{1+z}\;+b_{w}\left(-\varepsilon I_{0}S_{0}+M_{0}\left(-n_{p}+S_{0}\left(𝓋+2m_{p}+2b_{p}I_{0}+\omega_{p}-\delta_{p}\omega_{p}+\delta_{p}\omega_{p}^{\prime }\right)\right)\right)\;+A_{0}(-\sigma b_{w}S_{0}+2\kappa b_{p}^{2}I_{0}S_{0}-\kappa b_{p}(n_{p}-S_{0}(3m_{p}+2b_{w}M_{0}+\gamma_{p}+\omega_{p}-\delta_{p}\omega_{p}\;+\delta_{p}\omega_{p}^{\prime }))))+\cdots ,$$

$$I_{p}\left(t\right)=\overset{\infty }{\underset{n=0}{\sum }}{I_{p}}_{n}\left(t\right)=I_{0}+{I_{p}}_{1}\left(t\right)+{I_{p}}_{2}\left(t\right)+\cdots \;=I_{0}+\frac{t^{\alpha }}{\Gamma \left[\alpha +1\right]}\left(-I_{0}\left(m_{p}+\gamma_{p}\right)-E_{0}\left(-1+\delta_{p}\right)\omega_{p}\right)+\frac{t^{2\alpha }}{\Gamma \left[1+2\alpha \right]}(-\left(m_{p}+\gamma_{p}\right)(-I_{0}(m_{p}\;+\gamma_{p})-E_{0}\left(-1+\delta_{p}\right)\omega_{p})+\left(1-\delta_{p}\right)\omega_{p}(-m_{p}E_{0}+\kappa A_{0}b_{p}S_{0}+b_{w}M_{0}S_{0}+b_{p}I_{0}S_{0}\;+E_{0}\left(-1+\delta_{p}\right)\omega_{p}-E_{0}\delta_{p}\omega_{p}^{\prime }))+\cdots ,$$

$$A_{p}\left(t\right)=\overset{\infty }{\underset{n=0}{\sum }}{A_{p}}_{n}\left(t\right)=A_{0}+{A_{p}}_{1}\left(t\right)+{A_{p}}_{2}\left(t\right)+\cdots \;=A_{0}+\frac{t^{\alpha }}{\Gamma \left[\alpha +1\right]}\left(-A_{0}\left(m_{p}+\gamma_{p}\right)+E_{0}\delta_{p}\omega_{p}^{\prime }\right)+\frac{t^{2\alpha }}{\Gamma \left[1+2\alpha \right]}(-\delta_{p}\omega_{p}^{\prime }(2m_{p}E_{0}-b_{w}M_{0}S_{0}\;-b_{p}I_{0}S_{0}+E_{0}\gamma_{p}+E_{0}\omega_{p}-E_{0}\delta_{p}\omega_{p}+E_{0}\delta_{p}\omega_{p}^{\prime })+A_{0}(m_{p}^{2}+2m_{p}\gamma_{p}+\gamma_{p}^{2}\;+\kappa b_{p}S_{0}\delta_{p}\omega_{p}^{\prime }))+\cdots ,$$

$$R_{p}\left(t\right)=\overset{\infty }{\underset{n=0}{\sum }}{R_{p}}_{n}\left(t\right)=R_{0}+{R_{p}}_{1}\left(t\right)+{R_{p}}_{2}\left(t\right)+\cdots \;=R_{0}+\frac{t^{\alpha }}{\Gamma \left[\alpha +1\right]}\left(-m_{p}R_{0}+I_{0}\gamma_{p}+A_{0}\gamma_{p}^{\prime }\right)+\frac{t^{2\alpha }}{\Gamma \left[1+2\alpha \right]}(m_{p}^{2}R_{0}-I_{0}\gamma_{p}^{2}-A_{0}\gamma_{p}^{1+z}\;-2m_{p}\left(I_{0}\gamma_{p}+A_{0}\gamma_{p}^{\prime }\right)+E_{0}\gamma_{p}\omega_{p}-E_{0}\gamma_{p}\delta_{p}\omega_{p}+E_{0}\gamma_{p}^{\prime }\delta_{p}\omega_{p}^{\prime })+\cdots ,$$

$$M\left(t\right)=\overset{\infty }{\underset{n=0}{\sum }}{M_{p}}_{n}\left(t\right)=M_{0}+M_{1}\left(t\right)+M_{2}\left(t\right)+\cdots \;=M_{0}+\frac{t^{\alpha }}{\Gamma \left[\alpha +1\right]}\left(\sigma A_{0}-\vartheta M_{0}+\varepsilon I_{0}\right)+\frac{t^{2\alpha }}{\mathrm{Gamma}\left[1+2\alpha \right]}(\vartheta^{2}M_{0}-\vartheta \varepsilon I_{0}-\varepsilon m_{p}I_{0}-\varepsilon I_{0}\gamma_{p}\;-\sigma A_{0}\left(\vartheta +m_{p}+\gamma_{p}\right)+\varepsilon E_{0}\omega_{p}-\varepsilon E_{0}\delta_{p}\omega_{p}+\sigma E_{0}\delta_{p}\omega_{p}^{\prime })+\cdots .$$

## 5. Results and Discussion

**Figure 1:**
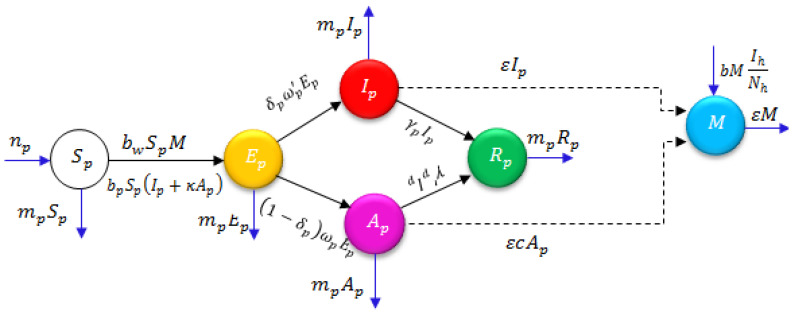
Flow chart of the above-described model [20].

We captured the behavior obtained for the projected model describing the 2019-nCoV epidemic with different fractional-order values. The considered model describes the outbreak of the coronavirus with the exponential increase in the number of people affected as it spreads. Figure 1 is a representation of the model, illustrating its evolution associated with each parameter. It is important to investigate the outbreak and its behavior with different parameters to analyze and predict its evolution and spread. The initial conditions considered for the present study, which include reported results from Wuhan, China [20,64], are:

$$S_{p}\left(0\right)=S_{0}=8,065,518,\;E_{p}\left(0\right)=E_{0}=200000,\;I_{p}\left(0\right)=I_{0}=282,\;A_{p}\left(0\right)=A_{0}=200,\;R_{p}\left(0\right)=R_{0}=0\;\mathrm{and}\;M\left(0\right)=M_{0}=50,000.$$

**Figure 2:**
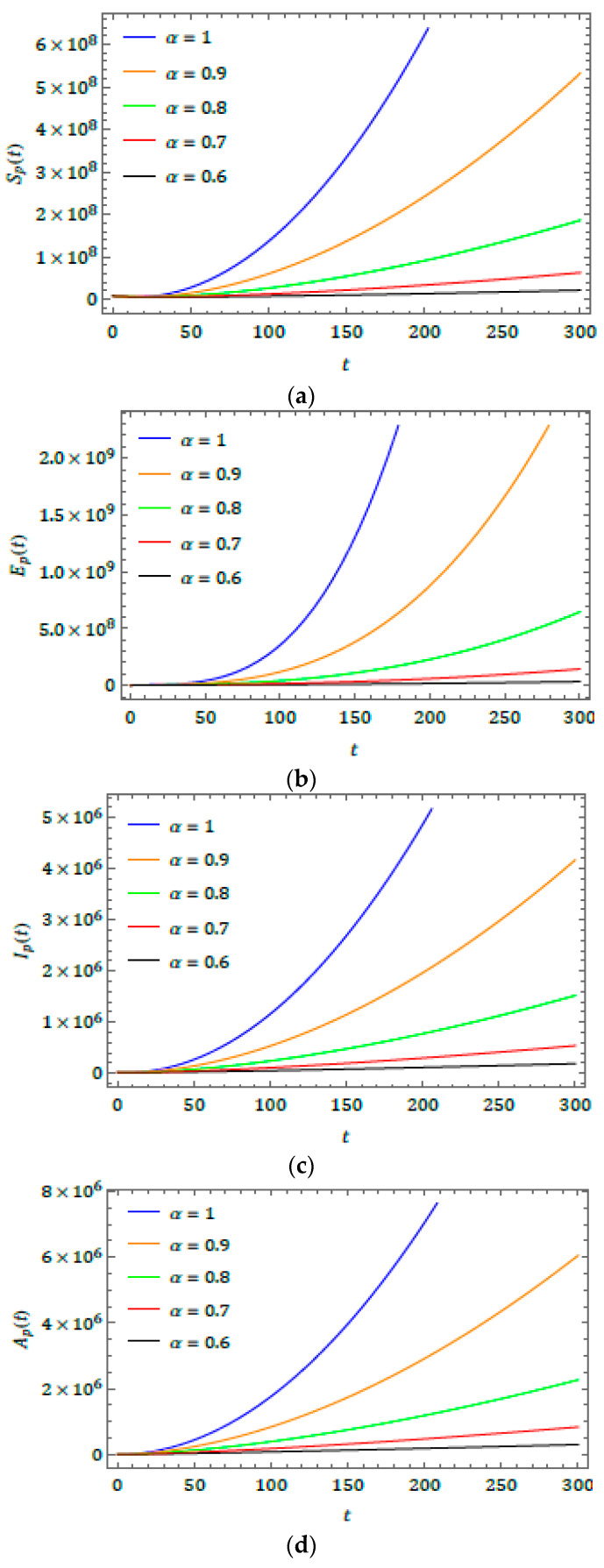

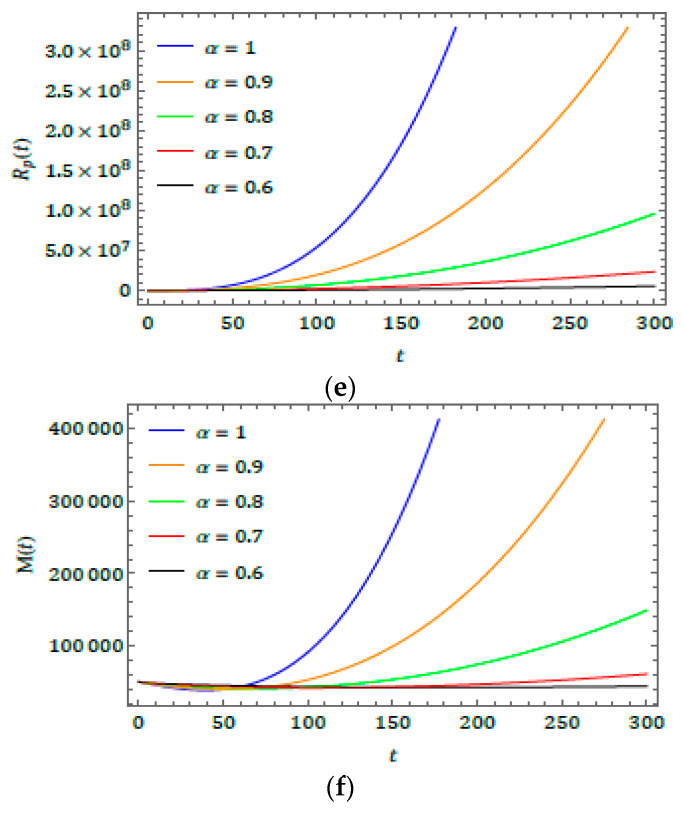
Behavior of results obtained for $\left(a\right)\;S_{p}\left(t\right),\left(b\right)E_{p}\left(t\right),\left(c\right)I_{p}\left(t\right),\left(d\right)A_{p}\left(t\right),\left(e\right)R_{p}\left(t\right)$, and (f) M(t) for distinct fractional order (α).

The present investigation may help researchers to understand some interesting consequences of the projected model. The fractional operator can also exemplify some future scenarios for the considered model, as shown in Figure 2. As the value of α changes, the obtained solution produces fascinating consequences, according to the fixed values of the parameters defined in the projected model. The plots show exponential growth in all classes, which corresponds to the spread of the virus from the beginning of 2020.

## 6. Conclusions

In this study, the fractional natural decomposition method was successfully applied to the investigation of 2019-nCoV, numerically illustrated by the spreading of some dependent variables of the 2019-nCoV system. Because the Caputo derivative and integral are recognized as suitable explanations of real-world problems, the present paper introduces the effectiveness of the considered derivative. Figure 1 explains how the transfer model occurs from reservoir to human. Figure 2 presents wave behaviors of infection and other features of the2019-nCoV outbreak. Thus, from Figure 1 and Figure 2, the results obtained using FNDM for 2019-nCoV are spreading shortly. We aimed to help researchers better understand the physical behavior of the novel coronavirus. The fractional-order method allows for more flexible investigations and deeper methods of observing2019-nCoV behaviors. The main novelty of this paper is that the simulation changes according to different fractional-order values. When the α value grows, the graphs notably increase. This explains 2019-nCoV spreading behaviors. Finally, we conclude that the projected method is extremely methodical, more effective, and very accurate, and can be applied to the analysis of many diverse classes of coupled nonlinear problems that exist in science and technology.

## References

[1] Chan J.F., Kok K.H., Zhu Z., Chu H., To K.K., Yuan S., Yuen K.Y. (2020). Genomic characterization of the 2019 novel human-pathogenic coronavirus isolated from patients with acute respiratory disease in Wuhan, Hubei, China. Emerg. Microbes Infect..

[2] Lu H., Stratton C.W., Tang Y.W. (2020). Outbreak of Pneumonia of Unknown Etiology in Wuhan China: The Mystery and the Miracle. J. Med. Virol..

[3] Ji W., Wang W., Zhao X., Zai J., Li X. (2020). Homologous recombination within the spike glycoprotein of the newly identified coronavirus may boost cross-species transmission from snake to human. J. Med. Virol..

[4] World Health Organization (2020). Coronavirus Disease 2019 (COVID-19) Situation Report-62. https://www.who.int/emergencies/diseases/novel-coronavirus-2019/situation-reports.

[5] Chen Y., Guo D. (2016). Molecular mechanisms of coronavirus RNA capping and methylation. Virol. Sin..

[6] Wang L.F., Shi Z., Zhang S., Field H., Daszak P., Eaton B.T. (2006). Review of bats and SARS. Emerg. Infect. Dis..

[7] Ge X.Y., Li J.L., Yang X.L., Chmura A.A., Zhu G., Epstein J.H., Mazet J.K., Hu B., Zhang W., Peng C. (2013). Isolation and characterization of a bat SARS-like coronavirus that uses the ACE2 receptor. Nature.

[8] Lu R., Zhao X., Li J., Niu P., Yang B., Wu H., Wang W., Song H., Huang B., Zhu N. (2020). Genomic characterisation and epidemiology of 2019 novel coronavirus: Implications for virus origins and receptor binding. Lancet.

[9] Zhou P., Yang X.-L., Wang X.G., Hu B., Zhang L., Zhang W., Si H.-R., Zhu Y., Li B., Huang C.-L. (2020). A pneumonia outbreak associated with a new coronavirus of probable bat origin. Nature.

[10] Tian X., Li C., Huang A., Xia S., Lu S., Shi Z., Lu L., Jiang S., Yang Z., Wu Y. (2020). Potent binding of 2019 novel coronavirus spike protein by a SARS coronavirus-specific human monoclonal antibody. Emerg. Microbes Infect..

[11] Ahmed S.F., Quadeer A.A., McKay M.R. (2020). Preliminary identification of potential vaccine targets for 2019-nCoV based on SARS-CoV immunological studies. Viruses.

[12] Rambaut A. (2020). Phylogenetic Analysis of 23 nCoV-2019 Genomes. http://virological.org/t/phylogenetic-analysis-of-23-ncov-2019-genomes-2020-01-23/335.

[13] Poon L.L.M., Chu D.K.W., Chan K.H., Wong O.K., Ellis T.M., Leung Y.H.C., Lau S.K.P., Woo P.C.Y., Suen K.Y., Yuen K.-Y. (2005). Identification of a novel coronavirus in bats. J. Virol..

[14] Chaudhury S., Berrondo M., Weitzner B.D., Muthu P., Bergman H., Gray J.J. (2011). Benchmarking and analysis of protein docking performance in Rosetta. PLoS ONE.

[15] Althaus C.L. (2015). Ebola super spreading. Lancet Infect. Dis..

[16] Huang C., Wang Y., Li X., Ren L., Zhao J., Hu Y., Zhang L., Fan G., Xu J., Gu X. (2020). Clinical features of patients infected with 2019 novel coronavirus in Wuhan, China. Lancet.

[17] Benvenuto D., Giovannetti M., Ciccozzi A., Spoto S., Angeletti S., Ciccozzi M. (2020). The 2019-new coronavirus epidemic: Evidence for virus evolution. J. Med. Virol..

[18] Ferguson N.M., Cummings D.A., Fraser C., Cajka J.C., Cooley P.C., Burke D.S. (2006). Strategies for mitigating an influenza pandemic. Nature.

[19] Koonin L.M., Cetron M.S. (2009). School closure to reduce influenza transmission. Emerg. Infect. Dis..

[20] Chen T.M., Rui J., Wang Q.P., Zhao Z.Y., Cui J.A., Yin L. (2020). A mathematical model for simulating the phase-based transmissibility of a novel coronavirus. Infect. Dis. Poverty.

[21] Mittag-Leffler G.M. (1903). Sur la nouvelle function E_α_(x). C. R. Acad. Sci. Paris.

[22] Khan Z.H., Khan W.A. (2008). N-Transform-Properties and Applications. NUST J. Eng. Sci..

[23] Loonker D., Banerji P.K. (2013). Solution of fractional ordinary differential equations by natural transform. Int. J. Math. Eng. Sci..

[24] Adomian G. (1984). A new approach to nonlinear partial differential equations. J. Math. Anal. Appl..

[25] Brzeziński D.W. (2018). Review of numerical methods for NumILPT with computational accuracy assessment for fractional calculus. Appl. Math. Nonlinear Sci..

[26] Rawashdeh M.S. (2017). The fractional natural decomposition method: Theories and applications. Math. Meth. Appl. Sci..

[27] Rawashdeh M.S., Maitama S. (2017). Finding exact solutions of nonlinear PDEs using the natural decomposition method. Math. Meth. Appl. Sci..

[28] Prakasha D.G., Veeresha P., Rawashdeh M.S. (2019). Numerical solution for (2 + 1)-dimensional time-fractional coupled Burger equations using fractional natural decomposition method. Math. Meth. Appl. Sci..

[29] Veeresha P., Prakasha D.G., Singh J. (2019). Solution for fractional forced KdV equation using fractional natural decomposition method. AIMS Math..

[30] Rawashdeh M.S. (2019). Solving fractional ordinary differential equations using FNDM. Thai J. Math..

[31] Prakasha D.G., Veeresha P., Baskonus H.M. (2019). Two novel computational techniques for fractional Gardner and Cahn-Hilliard equations. Comput. Math. Methods.

[32] Veeresha P., Prakasha D.G. (2020). An efficient technique for two-dimensional fractional order biological population model. Int. J. Model. Simul. Sci. Comput..

[33] Gao W., Veeresha P., Prakasha D.G., Baskonus H.M., Yel G. (2020). New numerical results for the time-fractional Phi-four equation using a novel analytical approach. Symmetry.

[34] Kumar D., Singh J., Baleanu D. (2018). Analysis of regularized long-wave equation associated with a new fractional operator with Mittag-Leffler type kernel. Physica A.

[35] Veeresha P., Baskonus H.M., Prakasha D.G., Gao W., Yel G. (2020). Regarding new numerical solution of fractional Schistosomiasis disease arising in biological phenomena. Chaos Solitons Fractals.

[36] Yokus A. (2018). Numerical solution for space and time fractional order Burger type equation. Alexandria Eng. J..

[37] Yang X.J., Gao F. (2017). A new technology for solving diffusion and heat equations. Therm. Sci..

[38] Atangana A., Baleanu D. (2016). New fractional derivatives with nonlocal and non-singular kernel theory and application to heat transfer model. Therm. Sci..

[39] Esen A., Tasbozan O. (2015). Cubic B-spline collocation method for solving time fractional gas dynamics equation. Tbilisi Math. J..

[40] Singh J., Kumar D., Baleanu D., Rathore S. (2019). On the local fractional wave equation in fractal strings. Math. Meth. Appl. Sci..

[41] Kaya D., Gulbahar S., Yokus A. (2018). Numerical solutions of the fractional KdV-Burgers-Kuramoto Equation. Therm. Sci..

[42] Atangana A., Alkahtani B.T. (2015). Analysis of the Keller-Segel model with a fractional derivative without singular kernel. Entropy.

[43] Goyal M., Baskonus H.M., Prakash A. (2019). An efficient technique for a time fractional model of lassa hemorrhagic fever spreading in pregnant women. Eur. Phys. J. Plus.

[44] Kumar D., Singh J., Baleanu D. (2018). A new numerical algorithm for fractional Fitzhugh-Nagumo equation arising in transmission of nerve impulses. Nonlinear Dyn..

[45] Yang X.J., Baleanu D., Lazarević M.P., Cajić M.S. (2015). Fractal boundary value problems for integral and differential equations with local fractional operators. Therm. Sci..

[46] Atangana A., Alkahtani B.T. (2016). Analysis of non- homogenous heat model with new trend of derivative with fractional order. Chaos Solitons Fractals.

[47] Yang X.J. (2018). New rheological problems involving general fractional derivatives with nonsingular power-law kernels. Proc. Rom. Acad. Ser. A Math. Phys. Tech. Sci. Inf. Sci..

[48] Yang X.J., Gao F., Ju Y., Zhou H.W. (2018). Fundamental solutions of the general fractional-order diffusion equations. Math. Meth. Appl. Sci..

[49] Kumar D., Singh J., Al-Qurashi M., Baleanu D. (2019). A new fractional SIRS-SI malaria disease model with application of vaccines, anti-malarial drugs, and spraying. Adv. Diff. Equ..

[50] Atangana A. (2020). Fractional discretization: The African’s tortoise walk. Chaos Solitons Fractals.

[51] Shah K., Khalil H., Khan R.A. (2018). Analytical solutions of fractional order diffusion equations by natural transform method. Iran. J. Sci. Technol. Trans. A Sci..

[52] Shah K., Junaid M., Ali N. (2015). Extraction of Laplace, Sumudu, Fourier and Mellin Transform from the Natural Transform. J. Appl. Environ. Biol. Sci..

[53] Shah K., Khan R.A. (2015). The applications of natural transform to the analytical solutions of some fractional order ordinary differential equations. Sindh Univ. Res. J. (Sci. Ser.).

[54] Shah K., Jarad F., Abdeljawad T. (2020). On a nonlinear fractional order model of dengue fever disease under Caputo-Fabrizio derivative. Alexandria Eng. J..

[55] Al-Ghafri K.S., Rezazadeh H. (2019). Solitons and other solutions of (3+1)-dimensional space–time fractional modified KdV–Zakharov–Kuznetsov equation. Appl. Math. Nonlinear Sci..

[56] Shah K., Alqudah M.A., Jarad F., Abdeljawad T. (2020). Semi-analytical study of Pine Wilt Disease model with convex rate under Caputo–Fabrizio fractional order derivative. Chaos Solitons Fractals.

[57] Khan A., Abdeljawad T., Gómez-Aguilar J.F., Khan H. (2020). Dynamical study of fractional order mutualism parasitism food web module. Chaos SolitonsFractals.

[58] Sene N. (2020). Stability analysis of electrical RLC circuit described by the Caputo-Liouville generalized fractional derivative. Alexandria Eng. J..

[59] Yokus A., Gulbahar S. (2019). Numerical solutions with linearization techniques of the fractional Harry Dym equation. Appl. Math. Nonlinear Sci..

[60] Sene N. (2020). Second-grade fluid model with Caputo–Liouville generalized fractional derivative. Chaos Solitons Fractals.

[61] Thiao A., Sene N. Fractional optimal economic control problem described by the generalized fractional order derivative. Proceedings of the International Conference on Computational Mathematics and Engineering Sciences (CMES 2019).

[62] Ionescu C., Lopes A., Copot D., Machado J.A.T., Bates J.H.T. (2017). The role of fractional calculus in modeling biological phenomena: A review. Commun. Nonlinear Sci. Numer. Simul..

[63] Qureshi S. (2020). Real life application of Caputo fractional derivative for measles epidemiological autonomous dynamical system. Chaos Solitons Fractals.

[64] Khan M.A., Atangana A. (2020). Modeling the dynamics of novel coronavirus (2019-nCov) with fractional derivative. Alexandria Eng. J..

[65] Bulut H., Baskonus H.M. (2016). Regarding on the prototype solutions for the nonlinear fractional-order biological population model. AIP Conf. Proc..

[66] Gao W., Veeresha P., Prakasha D.G., Baskonus H.M., Yel G. (2020). New approach for the model describing the deathly disease in pregnant women using Mittag-Leffler function. Chaos Solitons Fractals.

[67] Bulut H., Kumar D., Singh J., Swroop R., Baskonus H.M. (2018). Analytic study for a fractional model of HIV infection of CD4+TCD4+T lymphocyte cells. Math. Nat. Sci..

[68] Ilhan O.A., Esen A., Bulut H., Baskonus H.M. (2019). Singular Solitons in the Pseudo-parabolic Model Arising in Nonlinear Surface Waves. Results Phys..

